# Obinutuzumab-induced acute thrombocytopenia and leukopenia in ANCA-associated glomerulonephritis: case report and literature review

**DOI:** 10.3389/fimmu.2026.1845580

**Published:** 2026-05-05

**Authors:** Yiming Jin, Yiqi Huang, Xiaomiao He

**Affiliations:** 1 Department of Nephrology, Shaoxing Second Hospital, Shaoxing, Zhejiang, China; 2 Blood Purification Center, Shaoxing Second Hospital, Shaoxing, Zhejiang, China

**Keywords:** acute thrombocytopenia, anti-neutrophil cytoplasmic antibody-associated glomerulonephritis, case report, leukopenia, obinutuzumab

## Abstract

Obinutuzumab is a humanized type II anti-CD20 monoclonal antibody with enhanced antibody-dependent cellular cytotoxicity. It is widely used in B-cell malignancies and is increasingly being explored in autoimmune diseases such as lupus nephritis. The main adverse effects include infusion reactions and cytopenias. Among these, obinutuzumab-induced acute thrombocytopenia (OIAT) is a rare but potentially life-threatening complication. However, OIAT has been reported almost exclusively in patients with hematologic malignancies; data in autoimmune diseases remain limited. We report a 55-year-old male with anti-neutrophil cytoplasmic antibody-associated glomerulonephritis who developed acute severe thrombocytopenia and leukopenia shortly after the second dose of obinutuzumab, which was given due to rituximab intolerance. His platelet count (PLT) dropped from 121 × 10^9^/L to 2.5 × 10^9^/L, and white blood cell count (WBC) fell to 0.6 × 10^9^/L; the nadir occurred on day 3 after readmission. WBC recovered within one week, whereas PLT recovery took 45 days. To our knowledge, this is the first reported case of obinutuzumab-induced acute bicytopenia in an autoimmune disease setting. This case highlights the need for heightened awareness of hematologic toxicity when using obinutuzumab in patients with autoimmune diseases, and suggests that a rapid decline in PLT, even when still within the normal range, may serve as an early warning sign.

## 1 Introduction

Anti-neutrophil cytoplasmic antibody (ANCA) -associated vasculitis is an autoimmune disorder characterized by necrotizing inflammation of small vessels (1). The kidney is one of the most frequently affected target organs, and patients often present with rapidly progressive glomerulonephritis (RPGN) (2). Without timely intervention, the disease can progress to end-stage renal disease within weeks to months (3). For decades, the standard induction regimen for ANCA-associated glomerulonephritis has been glucocorticoids combined with cyclophosphamide. However, the risks of infection, gonadal toxicity, and long-term malignancy associated with this regimen remain persistent clinical challenges (4). As the central role of B cells in the pathogenesis of AAV has become better understood, rituximab (RTX) has gradually moved into first-line therapy for ANCA-associated glomerulonephritis (5, 6).

Obinutuzumab is a second-generation humanized type II anti-CD20 monoclonal antibody. Its Fc region has been glycoengineered to achieve higher affinity for FcγRIIIa, resulting in stronger antibody-dependent cell-mediated cytotoxicity (ADCC) and direct cell death effects, while complement-dependent cytotoxicity is relatively reduced (7, 8). This property makes obinutuzumab more effective than RTX in the treatment of B-cell lymphomas (9), and in recent years it has also been explored for autoimmune diseases such as lupus nephritis (10). However, stronger immune activation also brings new safety challenges. The incidence of obinutuzumab-induced acute severe thrombocytopenia (OIAT) is approximately 2%-3% (11), and the mechanism may involve complement activation (12, 13), Fcγ receptor-mediated clearance (12), and drug-dependent antibodies (DDA) (14). Reported cases of OIAT have occurred almost exclusively in patients with hematologic malignancies and most often present as isolated thrombocytopenia. In contrast, experience with obinutuzumab in ANCA-associated glomerulonephritis and other autoimmune diseases is limited, and obinutuzumab-induced hematologic toxicity has rarely been described.

We report a 55-year-old male with ANCA-associated glomerulonephritis who developed life-threatening acute severe thrombocytopenia along with leukopenia shortly after induction therapy with glucocorticoids plus obinutuzumab. To the best of our knowledge, this is the first case of obinutuzumab-induced acute bicytopenia occurring in the setting of an autoimmune disease. This report aims to alert clinicians to this rare but serious adverse reaction and to discuss its potential mechanisms and management.

## 2 Case presentation

**Figure 1:**
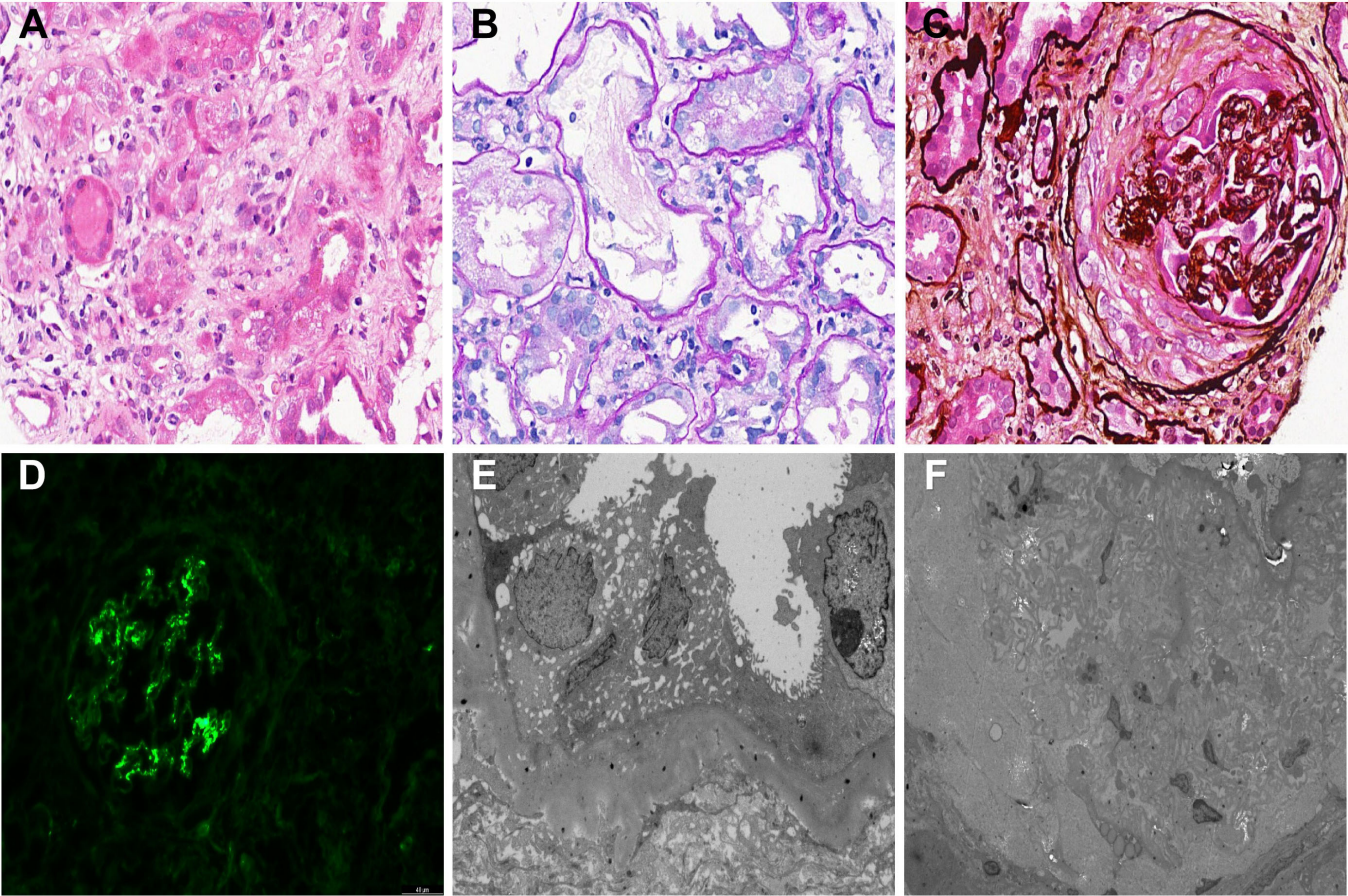
Kidney pathological findings of ANCA-associated vasculitis with membranous nephropathy. **(A)** HE staining: interstitial inflammatory infiltration, arteriolar wall thickening and luminal stenosis (×400). **(B)** PAS staining: glomerular capillary tuft collapse, tubular atrophy and interstitial fibrosis (×400). **(C)** PASM staining: thickened and wrinkled glomerular basement membrane with segmental spikes, crescents and global sclerosis (×400). **(D)** immunofluorescence: granular and nodular IgG and C3 deposits along capillary loops and mesangium (×200). **(E)** electron microscopy: reduced mitochondria, increased vacuoles and partial brush border loss in tubular epithelial cells (×2000). **(F)** electron microscopy: interstitial fibrosis with inflammatory infiltration, arteriolar smooth muscle hyperplasia and stenosis (×1000).

A 55-year-old man was admitted to our hospital on January 1, 2026, due to a two-month history of rising serum creatinine (SCr). A health checkup in November 2025 had revealed SCr 110 μmol/L and urine protein (UP) 1+, but the patient did not seek further evaluation or treatment. His condition gradually progressed, and he subsequently developed light red gross hematuria. He had no prior history of autoimmune or hematologic diseases. On admission, his vital signs were stable, with blood pressure 148/84 mmHg. Physical examination showed severe edema in both lower limbs. Laboratory findings were as follows: SCr 600 μmol/L, 24-hour UP 4.6 g, hemoglobin (Hb) 69 g/L, white blood cell count (WBC) 5.4 × 10^9^/L, absolute neutrophil count (ANC) 6.89 × 10^9^/L, platelet count (PLT) 227×10^9^/L, perinuclear ANCA 1:160, and anti-MPO antibody IgG 75.2 AU/ml. Based on these findings, the patient was diagnosed with ANCA-associated glomerulonephritis. He was started on empirical treatment with methylprednisolone 40 mg once daily. On January 3, 2026, an ultrasound-guided left kidney biopsy was performed. Light microscopy revealed 14 glomeruli, of which three were globally sclerosed and seven were approaching global sclerosis, accompanied by cellular, cellular fibrocellular and fibrous crescent formation, glomerular capillary tuft collapse and wrinkling, segmental mesangial hyperplasia, and subepithelial eosinophilic deposits. PASM staining demonstrated mild thickening and wrinkling of the glomerular basement membrane with segmental spike formation, which is the characteristic histopathological feature of membranous nephropathy. Tubulointerstitial injury included tubular atrophy (approximately 40%), epithelial degeneration, necrosis and shedding, protein casts and interstitial inflammatory infiltration. Small renal arteries showed wall thickening and smooth muscle hyperplasia with segmental stenosis. Congo red staining was negative. Immunofluorescence examination of 10 glomeruli showed IgG (++), C3 (+++), κ (+), λ (+) and IgG2 (++), with granular and nodular deposits along the capillary loops and mesangial area, which is typical immunofluorescence pattern of membranous nephropathy. IgA, IgM, C4, C1q, IgG1, IgG3, IgG4, PLA2R, Alb, HBcAg and HBsAg were all negative. Combining the clinical data with light microscopy, immunofluorescence findings, the characteristic spike formation on PASM staining, granular capillary loop deposits of IgG and C3, IgG2 subclass dominance and negative PLA2R, the pathological diagnosis was ANCA-associated glomerulonephritis with secondary membranous nephropathy (**Figure 1**). On January 5, 2026, the patient received methylprednisolone pulse therapy (500 mg daily for three days) combined with RTX (1 g × 2 doses). However, he developed severe chills and fever during the first infusion of RTX, which was considered intolerance. The infusion was stopped after 13 minutes, with a total administered dose of less than 10 mg. Therefore, the treatment was switched to obinutuzumab 1 g on January 9, 2026. At that time, PLT was 272×10^9^/L. In addition, he underwent three sessions of plasma exchange (PE) on January 13, 16, and 19. On January 13, PLT was 182×10^9^/L. On January 23, 2026, a second dose of obinutuzumab 1 g was administered, and PLT was 121×10^9^/L, Hb 109 g/L, and SCr 375 μmol/L. After treatment, the patient’s hematuria resolved and renal function improved. Considering his condition was under control, he was discharged on January 24, 2026.

**Figure 2:**
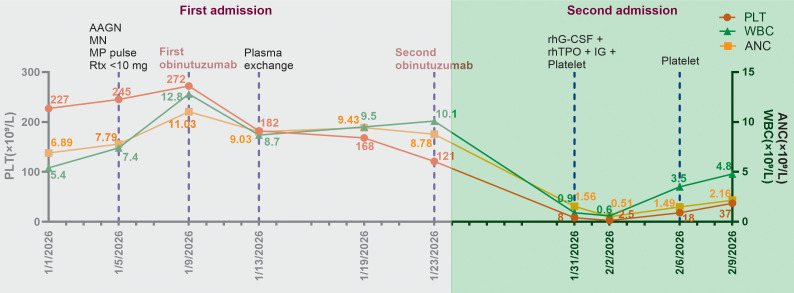
The timeline for diagnosis and treatment.

Seven days after discharge, the patient developed dark red gross hematuria. He came to our emergency department, where laboratory tests showed PLT 8×10^9^/L, WBC 0.9×10^9^/L, ANC 1.56×10^9^/L, and Hb 77 g/L. Given the rapid decline in WBC, ANC and PLT together with other findings, we suspected bone marrow suppression induced by biologic agents. He was readmitted and immediately started on recombinant human granulocyte colony-stimulating factor (rhG-CSF, 300 μg, subcutaneously once daily), recombinant human thrombopoietin (rhTPO, 15000 U subcutaneously once daily), and immunoglobulin (IG, 5 g intravenously once daily), along with platelet transfusions (1.5 units intravenously once daily) for two consecutive days. On hospital day 3, the patient’s WBC (0.6×10^9^/L), ANC (0.51×10^9^/L), and PLT (2.5×10^9^/L) simultaneously reached their nadir, while Hb remained stable (85 g/L). On day 4, gross hematuria began to improve. By day 7, the hematuria had completely resolved, WBC returned to normal (3.5×10^9^/L), and rhG-CSF was discontinued; ANC and PLT had increased modestly, and another unit of platelets was transfused. On hospital day 10, ANC returned to the normal range and PLT rose further to 37×10^9^/L. The patient’s condition was stable, and he was discharged for outpatient follow-up. During the hospital stay, Hb remained within 77–90 g/L without significant fluctuation or decline. On continued follow-up after discharge, PLT returned to normal (162×10^9^/L) on day 45. The diagnostic and treatment timeline is summarized in **Figure 2**.

## 3 Discussion

The use of obinutuzumab in ANCA-associated glomerulonephritis is still in the exploratory stage. For instance, in lupus nephritis, a phase II trial and its subsequent phase III study have shown that obinutuzumab can improve renal response rates, but the incidence of hematologic adverse events was significantly higher than in the placebo group (10). In contrast, no prospective data are available for ANCA-associated glomerulonephritis, and only case reports have described its use in patients who were intolerant or refractory to RTX (11, 15–17). To our knowledge, this is the first report of obinutuzumab-induced acute thrombocytopenia combined with leukopenia in an autoimmune disease setting, and the timing and pattern of cytopenias in our case differ markedly from previously reported cases of OIAT. This case highlights the need for a high index of suspicion for hematologic toxicity when using obinutuzumab in such patients.

Before blaming obinutuzumab for the bicytopenia, we ruled out other common causes. Blood cultures were negative, the patient had no fever or systemic inflammation, and procalcitonin and CRP were normal, making infection unlikely. Peripheral blood smear showed no schistocytes, LDH was normal, direct antiglobulin test negative, and no new renal or neurological symptoms appeared beyond baseline, so thrombotic microangiopathy was unlikely. Coagulation tests were normal, excluding DIC. As for prior treatments, the patient had received only a low maintenance dose of methylprednisolone (40 mg/day) plus a short 3−day pulse of 500 mg, which rarely causes severe bicytopenia. Three sessions of plasma exchange were finished more than a week before the nadir, and plasma exchange alone does not explain delayed marrow suppression. The earlier rituximab dose was less than 10 mg because of intolerance, far below the usual dose that causes cytopenia. Therefore, obinutuzumab remained the most likely cause.

To date, only 16 well-documented cases of obinutuzumab-induced acute thrombocytopenia (OIAT) have been reported in the literature, all occurring in patients with hematologic malignancies (11, 12, 14, 15, 18–23). Our case represents the first instance of OIAT in the setting of an autoimmune disease, suggesting that the immunological effects of obinutuzumab may differ across disease contexts. The clinical characteristics of these 16 patients are summarized in **Table 1**. Among them, 68.8% (11/16) were female, the age ranged from 28 to 83 years, and follicular lymphoma accounted for 75.0% (12/16). Regarding clinical presentation, bleeding symptoms were clearly documented in 56.3% (9/16), most commonly petechiae, ecchymoses, and epistaxis; only one patient (6.3%) had mucosal bleeding, while the remaining cases had no overt bleeding. All previously reported OIAT cases involved isolated thrombocytopenia, whereas our patient also developed severe leukopenia (nadir 0.6×10^9^/L), suggesting that obinutuzumab may induce broader myeloid lineage destruction. In terms of timing, 81.3% (13/16) of cases experienced a sharp drop in PLT within 24 h of the first infusion, with the case reported by Sakai et al. showing a fall as early as 1 h after infusion (18); only 18.7% (3/16) had onset several days to more than ten days later (11, 18, 21). PLT fell below 20×10^9^/L in all patients, and in 50.0% (8/16) it dropped below 5×10^9^/L. Among the 12 patients with documented re-exposure, 66.7% (8/12) experienced recurrence; in the case reported by Kou et al., PLT fell to 1-2×10^9^/L upon re-exposure, which was more severe than the initial episode (12).

**Table 1:** Summary of obinutuzumab-induced acute thrombocytopenia cases.

References	Disease	Age/Sex	PLT (×10^9^/L)		Onset relative to obinutuzumab	Clinical presentation	Rechallenge outcome	Treatment	Recovery time	Unique OIAT feature
			Baseline	Nadir						
Walter et al. (20)	CLL	68/F	Unclear	Grade 3	Within 30 min of first infusion	Minor epistaxis	Subsequent infusions uneventful	Supportive, platelets	Unclear	First OIAT with DIC
Sakai et al. (18)	FL	64/F	211	21	1h post-infusion; nadir at day 4	No bleeding	Recurred on maintenance	Platelet transfusion	10 days	Detailed kinetics: 1h drop, 6h half, 4d nadir
Haage et al. (19)	FL	56/F	245	4	Within 24h of cycle 1	No bleeding	Not rechallenged	IVIg, platelets	6 days	IVIg rapidly effective
Mechelfekh et al. (15)	FL	74/F	376	3	Within 1 day of cycle 1	Epistaxis, petechial purpura of limbs, bruises	Milder after switch to R	Platelets, romiplostim	6 days	FDA database: 62 serious cases, 6 deaths
	MCL	44/F	76	3	Within 1 day (pre−treatment)	Unclear	Worse on re−exposure	Platelets, eltrombopag	Weeks	TPO-RA associated DVT
Ng et al. (11)	CLL	83/F	107	4	After cycle 3	No bleeding	Rapid drop on rechallenge	Stop obinutuzumab	Unclear	Real-world incidence 2.7%
	FL	66/F	208	14	Day 11 of cycle 1	Bleeding	Recurred on maintenance	IVIg, steroids	Unclear	Platelet transfusion ineffective; IVIg effective
	FL	74/M	221	13	Day 28 of cycle 1	Unclear	Recurred on maintenance	Stop obinutuzumab	10 weeks	Slow recovery (10 weeks)
	FL	82/M	103	39	Day 18 of cycle 1	No bleeding	Not rechallenged	Switch to R	Unclear	No recurrence after switching to R
Yilmaz et al. (22)	DLBCL	81/F	144	33	Within 36h of first dose	Unclear	No recurrence on subsequent doses	Platelet	6 days	No recurrence on subsequent doses
	FL	47/M	111	23	Day 2 of first dose	Mucosal bleeding	No recurrence on 8 maintenance doses	Platelet	Days	No recurrence on subsequent maintenance
	FL	41/M	112	13	Day 5 of first dose	Mild bleeding symptoms	No recurrence on subsequent O−ICE	Platelet	23 days	No recurrence despite continued obinutuzumab
Dou et al. (23)	FL	38/F	130	15	Cycle 1 day 2, cycle 2 day 1	No bleeding	Worse on second dose	Platelet	8d/29d	Extended interval or switch to R reduces recurrence risk
Kou et al. (12)	FL	28/M	191	1-2	Day 3 of maintenance (severe all 3 times)	Ecchymoses and petechiae on skin and mucous membranes	Severe each time	Steroids, IL−11, platelets	2 months	Proposed DDA mechanism; steroids + IL-11 > IL-11 alone
Tane et al. (14)	FL	65/F	222	11	Within 12h of cycle 1	Unclear	Not rechallenged	Platelets, eltrombopag	29 days	First lab evidence supporting platelet consumption
Zhou et al. (21)	FL	47/F	199	3	Within 24h after cycle 2	No bleeding	Recurred on continued use	rhTPO, platelets	23d to 80d	Only case with pre/post bone marrow biopsy showing megakaryocyte dysfunction

FL, follicular lymphoma; CLL, chronic lymphocytic leukemia; MCL, mantle cell lymphoma; DLBCL, diffuse large B-cell lymphoma; BM, bone marrow; DIC, disseminated intravascular coagulation; IPF, immature platelet fraction; TPO, thrombopoietin; PA-IgG, platelet-associated immunoglobulin G; IVIg, intravenous immunoglobulin; TPO−RA, thrombopoietin receptor agonist; DDA, drug−dependent antibody; R, rituximab; O-ICE, obinutuzumab, ifosfamide, carboplatin, etoposide; ASCT, autologous stem cell transplantation; DVT, deep vein thrombosis; OIAT, obinutuzumab-induced acute thrombocytopenia.

The treatment course and outcome in our case also differed from those of previously reported OIAT cases. After the patient developed severe bicytopenia, we promptly initiated supportive therapy with rhTPO, rhG-CSF, and IG. WBC returned to normal on hospital day 7, while PLT began to rise on day 7 and reached normal levels on day 45. Compared with previous cases, our patient showed a slower response and a longer recovery period. This difference may be attributable to the bicytopenic and delayed presentation, suggesting that the underlying mechanism may differ from the classic acute immune-mediated OIAT. Of note, obinutuzumab has been reported to induce T-cell functional changes and has been associated with macrophage activation syndrome (24), which might account for the delayed and more extensive immune-mediated cytopenias. Furthermore, among the five patients in **Table 1** who received IG, 80.0% (4/5) recovered within one week (12, 14, 19), whereas our patient recovered more slowly despite the use of IG. This further suggests that the pathophysiology of bicytopenia is more complex and that IG alone may not be sufficient for rapid reversal. rhG-CSF and rhTPO may support compensatory bone marrow proliferation by promoting the proliferation and differentiation of granulocytic and megakaryocytic progenitors (21). In addition, the clinical characteristics and outcomes of the reported OIAT cases were heterogeneous. Walter et al. described the first OIAT case complicated by disseminated intravascular coagulation (DIC) (20); Sakai et al. detailed the kinetics of PLT decline (18); Haage et al. reported rapid recovery within six days after IG (19); Mechelfekh et al., using FDA database analysis, identified 62 serious cases with six deaths and also reported a case of mantle cell lymphoma that developed deep vein thrombosis following treatment with a TPO receptor agonist (15); Ng et al. estimated the incidence of OIAT at approximately 2.7% (11); Yilmaz et al. described two patients who did not experience recurrence during subsequent maintenance therapy (22); Dou et al. found that extending the infusion interval or switching to RTX reduced the risk of recurrence (23); Kou et al. proposed a DDA mechanism (12); Tane et al. provided laboratory evidence supporting platelet consumption (14); and Zhou et al. reported the only case with bone marrow biopsy performed both before and after OIAT, showing megakaryocyte dysfunction (21). In contrast, our patient exhibited an acute cytopenic episode after the second infusion, with a markedly delayed onset that clearly differs from classic acute OIAT, suggesting a more complex pathogenesis. Notably, between the first and second obinutuzumab infusions, the patient’s PLT dropped from 272×10^9^/L to 122×10^9^/L, a 55.1% decrease. Although the absolute count remained within the normal range, this significant downward trend may serve as an early warning sign of subsequent fulminant thrombocytopenia. Thus, clinical monitoring should focus not only on whether PLT falls below the normal limit but also on the magnitude of change; a sharp decline over a short period, even within normal range, warrants heightened vigilance and close follow-up.

Another distinct feature of this case is severe renal impairment (baseline SCr 600 μmol/L), which we examined for potential impact on obinutuzumab pharmacokinetics or cytopenia risk. A phase 1b study by Redfield et al. tested obinutuzumab in end-stage renal disease patients and found it well tolerated, with most adverse events grade 1 or 2 (25). Renal impairment was not clearly linked to increased hematologic toxicity. Obinutuzumab is cleared mainly by non-renal pathways such as proteolytic degradation, so its pharmacokinetics are not affected by renal function. The phase III GOYA trial also confirmed no significant association between renal impairment and grade 3-4 cytopenias after obinutuzumab treatment (26). Meanwhile, Hartinger et al. pointed out that in nephrotic syndrome, proteinuria and other factors can alter pharmacokinetics and affect B-cell depletion (27). Thus, we cannot rule out that renal failure contributed to the severity or delayed recovery of bicytopenia in our patient. No large-scale study has specifically examined the safety of obinutuzumab in autoimmune patients with severe renal impairment. Therefore, while renal impairment may not directly alter obinutuzumab pharmacokinetics, it could still increase the risk of hematologic adverse events through indirect mechanisms. This unique aspect adds clinical value to our report and calls for greater vigilance when using obinutuzumab in patients with renal dysfunction.

Regarding mechanisms, several hypotheses have been proposed for obinutuzumab-induced cytopenia, with immune-mediated destruction considered central. The type II structure and glycoengineered Fc region of obinutuzumab confer higher affinity for FcγRIIIa on immune effector cells, thereby enhancing ADCC (28, 29). This potent immune activation may cause “collateral damage” to CD20-negative cells that become opsonized by the drug or complement. For platelets, obinutuzumab may opsonize them via C3b fragments generated through complement activation, leading to clearance by FcγR-expressing macrophages in the spleen (12). Neutrophils express FcγRIIIb on their surface, which can bind the Fc portion of obinutuzumab and theoretically trigger ADCC, providing a possible explanation for the bicytopenia observed in our case. Evidence also supports the involvement of DDA (18, 19, 30). In this mechanism, the drug acts as a bridge, inducing specific antibodies that recognize platelet membrane glycoproteins only in its presence, resulting in rapid platelet clearance. This aligns well with the rapid and severe thrombocytopenia upon re-exposure. However, the delayed presentation in our case is difficult to explain solely by a DDA mechanism requiring the presence of the drug, suggesting a more complex process such as delayed hypersensitivity or immune reconstitution. Additionally, bone marrow examinations in some OIAT cases have shown normal or increased megakaryocyte counts (15, 21), indicating that peripheral destruction rather than impaired marrow production is the predominant mechanism. This is consistent with the pattern observed in our case, where PLT and WBC declined simultaneously while Hb remained relatively stable, an “erythroid escape” pattern.

Based on the available evidence and our experience, we propose the following clinical recommendations. When obinutuzumab is used for autoimmune diseases, complete blood counts should be monitored dynamically for at least 4 weeks after infusion, with close attention to downward trends. A decline of more than 50% over a short period should be considered a high-risk signal, even if absolute counts remain within normal range. If unexplained cytopenia occurs, infectious causes, thrombotic microangiopathy, and disease activity should be excluded; bone marrow aspiration may help differentiate immune destruction from bone marrow failure. Once obinutuzumab is confirmed or strongly suspected as the cause, the drug should be discontinued immediately. RTX, owing to its distinct mechanism, may serve as a safe alternative for subsequent treatment. Re-challenge with obinutuzumab should be avoided given the high recurrence rate and potential for more severe cytopenias.

## 4 Conclusion

In conclusion, this is the first report of obinutuzumab-induced acute severe bicytopenia in a patient with ANCA-associated glomerulonephritis, highlighting a rare but life-threatening complication of this agent in autoimmune diseases. Based on this single case, a rapid decline in platelet count, even when values remain within the normal range, may serve as a potential early warning sign. Clinicians should be aware of the need for vigilant hematologic monitoring during obinutuzumab therapy in autoimmune disease patients, and further studies are warranted to confirm these observations and establish optimal management strategies.

## Data Availability

The original contributions presented in the study are included in the article/**Supplementary Material**. Further inquiries can be directed to the corresponding author.
