# Supplementary material for: Obinutuzumab-induced acute thrombocytopenia and leukopenia in ANCA-associated glomerulonephritis: case report and literature review
Source: Front Immunol. 2026 May 5;17:1845580. doi: 10.3389/fimmu.2026.1845580 (PMC13183652; doi:10.3389/fimmu.2026.1845580)
Supplement: Supplementary file 1 [file Table1.docx]

Supplementary Material

# Supplementary Figures and Tables

## Supplementary Figures


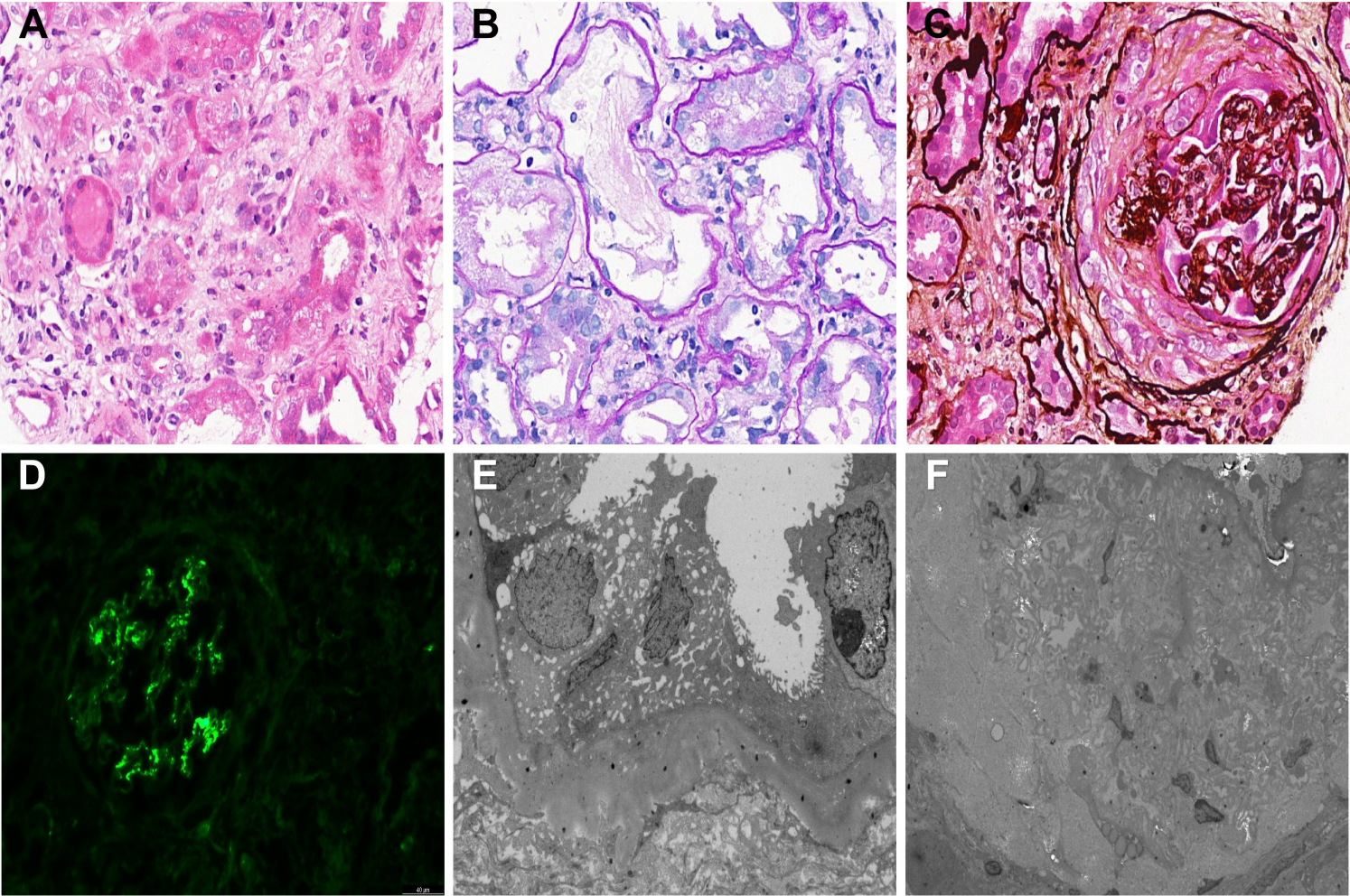


**Supplementary Figure 1.** Kidney pathological findings of ANCA-associated vasculitis with membranous nephropathy. (A) HE staining: Interstitial inflammatory infiltration, arteriolar wall thickening and luminal stenosis (×400). (B) PAS staining: Glomerular capillary tuft collapse, tubular atrophy and interstitial fibrosis (×400). (C) PASM staining: Thickened and wrinkled glomerular basement membrane with segmental spikes, crescents and global sclerosis (×400). (D) Immunofluorescence : Granular and nodular IgG and C3 deposits along capillary loops and mesangium (×200). (E) Electron microscopy: Reduced mitochondria, increased vacuoles and partial brush border loss in tubular epithelial cells (×2000). (F) Electron microscopy: Interstitial fibrosis with inflammatory infiltration, arteriolar smooth muscle hyperplasia and stenosis (×1000).

**
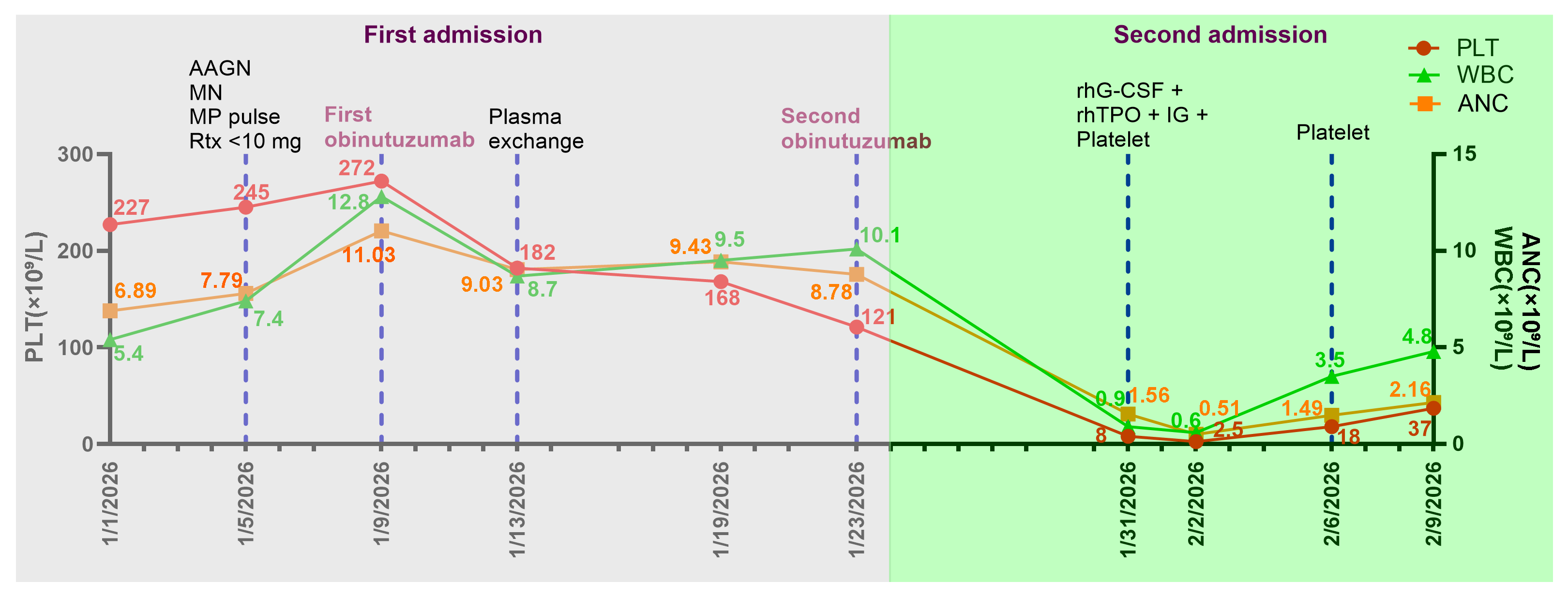
Supplementary Figure 2.** The timeline for diagnosis and treatment.

## Supplementary Tables

### **Supplementary Table 1.** Summary of Obinutuzumab-Induced Acute Thrombocytopenia Cases

| References | Disease | Age/Sex | PLT (×10⁹/L) | | Onset Relative to Obinutuzumab | Clinical Presentation | Rechallenge Outcome | Treatment | Recovery Time | Unique OIAT Feature |
| --- | --- | --- | --- | --- | --- | --- | --- | --- | --- | --- |
|  |  |  | Baseline | Nadir |  |  |  |  |  |  |
| Walter et al. (20) | CLL | 68/F | Unclear | Grade 3 | Within 30 min of first infusion | Minor epistaxis | Subsequent infusions uneventful | Supportive, platelets | Unclear | First OIAT with DIC |
| Sakai et al. (18) | FL | 64/F | 211 | 21 | 1h post-infusion; nadir at day 4 | No bleeding | Recurred on maintenance | Platelet transfusion | 10 days | Detailed kinetics: 1h drop, 6h half, 4d nadir |
| Haage et al. (19) | FL | 56/F | 245 | 4 | Within 24h of cycle 1 | No bleeding | Not rechallenged | IVIg , platelets | 6 days | IVIg rapidly effective |
| Mechelfekh et al. (15) | FL | 74/F | 376 | 3 | Within 1 day of cycle 1 | Epistaxis, petechial purpura of limbs, bruises | Milder after switch to R | Platelets, romiplostim | 6 days | FDA database: 62 serious cases, 6 deaths |
|  | MCL | 44/F | 76 | 3 | Within 1 day (pre‑treatment) | Unclear | Worse on re‑exposure | Platelets, eltrombopag | Weeks | TPO-RA associated DVT |
| Ng et al. (11) | CLL | 83/F | 107 | 4 | After cycle 3 | No bleeding | Rapid drop on rechallenge | Stop obinutuzumab | Unclear | Real-world incidence 2.7% |
|  | FL | 66/F | 208 | 14 | Day 11 of cycle 1 | Bleeding | Recurred on maintenance | IVIg, steroids | Unclear | Platelet transfusion ineffective; IVIg effective |
|  | FL | 74/M | 221 | 13 | Day 28 of cycle 1 | Unclear | Recurred on maintenance | Stop obinutuzumab | 10 weeks | Slow recovery (10 weeks) |
|  | FL | 82/M | 103 | 39 | Day 18 of cycle 1 | No bleeding | Not rechallenged | Switch to R | Unclear | No recurrence after switching to R |
| Yilmaz et al. (22) | DLBCL | 81/F | 144 | 33 | Within 36h of first dose | Unclear | No recurrence on subsequent doses | Platelet | 6 days | No recurrence on subsequent doses |
|  | FL | 47/M | 111 | 23 | Day 2 of first dose | Mucosal bleeding | No recurrence on 8 maintenance doses | Platelet | Days | No recurrence on subsequent maintenance |
|  | FL | 41/M | 112 | 13 | Day 5 of first dose | Mild bleeding symptoms | No recurrence on subsequent O‑ICE | Platelet | 23 days | No recurrence despite continued obinutuzumab |
| Dou et al. (23) | FL | 38/F | 130 | 15 | Cycle 1 day 2, cycle 2 day 1 | No bleeding | Worse on second dose | Platelet | 8d/29d | Extended interval or switch to R reduces recurrence risk |
| Kou et al. (12) | FL | 28/M | 191 | 1-2 | Day 3 of maintenance (severe all 3 times) | Ecchymoses and petechiae on skin and mucous membranes | Severe each time | Steroids, IL‑11, platelets | 2 months | Proposed DDA mechanism; steroids + IL‑11 > IL‑11 alone |
| Tane et al. (14) | FL | 65/F | 222 | 11 | Within 12h of cycle 1 | Unclear | Not rechallenged | Platelets, eltrombopag | 29 days | First lab evidence supporting platelet consumption |
| Zhou et al. (21) | FL | 47/F | 199 | 3 | Within 24h after cycle 2 | No bleeding | Recurred on continued use | rhTPO, platelets | 23d to 80d | Only case with pre/post bone marrow biopsy showing megakaryocyte dysfunction |

FL, follicular lymphoma; CLL, chronic lymphocytic leukemia; MCL, mantle cell lymphoma; DLBCL, diffuse large B-cell lymphoma; BM, bone marrow; DIC, disseminated intravascular coagulation; IPF, immature platelet fraction; TPO, thrombopoietin; PA-IgG, platelet-associated immunoglobulin G; IVIg, intravenous immunoglobulin; TPO‑RA, thrombopoietin receptor agonist; DDA, drug‑dependent antibody; R, rituximab; O-ICE, obinutuzumab, ifosfamide, carboplatin, etoposide; ASCT, autologous stem cell transplantation; DVT, deep vein thrombosis; OIAT, obinutuzumab-induced acute thrombocytopenia;
